# Motivations for active commuting: a qualitative investigation of the period of home or work relocation

**DOI:** 10.1186/1479-5868-9-109

**Published:** 2012-09-11

**Authors:** Caroline HD Jones, David Ogilvie

**Affiliations:** 1 MRC Epidemiology Unit and UKCRC Centre for Diet and Activity Research (CEDAR), Box 296, Institute of Public Health, Forvie Site, Robinson Way, Cambridge, CB2 OSR, UK; 2 MRC Epidemiology Unit and CEDAR, Cambridge. She is now at the Department of Primary Care Health Sciences, University of Oxford, Oxford, UK; 3 Department of Primary Care Health Sciences, University of Oxford, Radcliffe Observatory Quarter, Woodstock Road, Oxford, OX2 6GG, UK

**Keywords:** Active commuting, Qualitative, Relocation, Habit discontinuity, Residential self-selection

## Abstract

**Background:**

Promoting walking or cycling to work (active commuting) could help to increase population physical activity levels. According to the habit discontinuity and residential self-selection hypotheses, moving home or workplace is a period when people (re)assess, and may be more likely to change, their travel behavior. Research in this area is dominated by the use of quantitative research methods, but qualitative approaches can provide in-depth insight into the experiences and processes of travel behavior change. This qualitative study aimed to explore experiences and motivations regarding travel behavior around the period of relocation, in an effort to understand how active commuting might be promoted more effectively.

**Methods:**

Participants were recruited from the Commuting and Health in Cambridge study cohort in the UK. Commuters who had moved home, workplace or both between 2009 and 2010 were identified, and a purposive sample was invited to participate in semi-structured interviews regarding their experiences of, and travel behavior before and after, relocating. A grounded theory approach was taken to analysis.

**Results:**

Twenty-six commuters participated. Participants were motivated by convenience, speed, cost and reliability when selecting modes of travel for commuting. Physical activity was not a primary motivation, but incidental increases in physical activity were described and valued in association with active commuting, the use of public transport and the use of park-and-ride facilities.

**Conclusions:**

Emphasizing and improving the relative convenience, cost, speed and reliability of active commuting may be a more promising approach to promoting its uptake than emphasizing the health benefits, at least around the time of relocation. Providing good quality public transport and free car parking within walking or cycling distance of major employment sites may encourage the inclusion of active travel in the journey to work, particularly for people who live too far from work to walk or cycle the entire journey. Contrary to a straightforward interpretation of the self-selection hypothesis, people do not necessarily decide how they prefer to travel, relocate, and then travel in their expected way; rather, there is constant negotiation, reassessment and adjustment of travel behavior following relocation which may offer an extended window of opportunity for travel behavior change.

## Background

Promoting physical activity is a public health priority. It is recommended that adults should take part in ≥150 minutes of moderate intensity physical activity each week, but most adults in the UK do not achieve this [1]. One strategy for increasing population physical activity levels is to promote walking and cycling for transport (active travel). Commuter journeys account for the greatest distances travelled by UK adults [2], and are a way in which physical activity could be built into the daily routine; hence incorporating walking and cycling into the journey to work (active commuting) has been identified as a particular strategy to reduce physical inactivity [3]. Indeed, active commuting has been found to be associated with lower levels of overweight and obesity than car commuting [4,5], and also with reduced sickness absence [6], cardiovascular risk and mortality [7,8]. Yet levels of active commuting are low: in 2010, 70% of UK adults usually travelled to work by car, compared to 14% by walking or cycling [9]. Understanding the determinants of travel to work, and how levels of active commuting could be increased, is therefore important.

The term “active commuting” will be used throughout this paper to refer to those modes of travel to and from work which involve physical activity (walking and cycling). According to this definition, active commuting can involve commuting solely by walking or cycling; or by walking or cycling in combination with motorized modes of travel (for example a combination of car with walking, or of train with cycling). We use the term “inactive commuting” to refer to those modes of travel to and from work which do not involve any significant physical activity (motorized travel only). Inactive commuting can involve one or more inactive modes of travel.

An array of physical environmental, psychological and social factors interacts to determine (active) travel behaviour [10], but these do not fully explain individuals’ travel behaviour to work. In order to explore how active commuting can be promoted, it is useful to further examine how individuals select and change their travel modes. However, commuting is relatively resistant to change due to its habitual nature [11]. Habits become established when everyday activities such as commuting to work are performed repetitively and in stable contexts (in particular locations at specific times). Habitual behaviours are performed with little conscious intention: individuals with strong habits are less likely to acquire information about alternative options, and are resistant to reconsidering or changing behaviour [12,13].

The habit discontinuity hypothesis, described by Verplanken et al. [14], posits that behaviour change is more likely when habits are broken by a change of context. Disrupting the environmental cues which trigger habits can break them, leading relevant information to become more influential; in this window of opportunity following a context change, individuals are more likely to consciously reconsider, and perhaps change, their behaviour [12,15]. A naturally occurring context change which disrupts the habit of commuting to work is the relocation of home or workplace. Verplanken et al. tested the habit discontinuity hypothesis in this context, in combination with the self-activation hypothesis (which states that values influence behaviour when the value is part of a person’s self-concept and is cognitively activated) [14]. They compared travel behaviour and environmental concerns in university employees who had, or had not, moved home in the last year. As hypothesised, participants who had recently moved home and who expressed environmental concerns commuted to work by car less frequently than those who were environmentally concerned and had not recently moved (as well as less frequently than those who were low on environmental concern and had moved) [14]. This supports the assumption that context change (relocation), guided by activated values, can lead to negotiation of travel behaviour.

Relocation may also lead to travel behaviour change because the way in which people travel is associated with characteristics of the local built environment [16-18]. Indeed, researchers have examined residential relocation in an effort to better understand relationships between the built environment, attitudes and preferences, and travel behaviour [19,20]. The self-selection hypothesis proposes that households choose locations based on how they expect or prefer to travel; for example, people wishing to cycle will live in areas accessible and convenient for cycling, and will then travel by bike [19]. This sorting process may contribute to the observed associations between the built environment and travel behaviour.

Thus, the habit discontinuity and residential self-selection hypotheses both suggest that relocation is a period when travel choices may be considered and reviewed and travel behaviour may be more likely to change. Studying the period of relocation (of home or work) could therefore further our understanding of how and why people select or change their travel behaviour to work, which is important for informing the design of interventions to increase active commuting.

Research in this area is dominated by the use of quantitative research methods. For example, residential relocation studies have tended to focus on the quantitative assessment of travel behaviour and attitudes before and after relocation [20]. Few studies have used qualitative methods to explore active travel [21-24]. It is timely to reach beyond quantitative measures of travel behaviour, and quantitative estimates of associations between travel behaviour and the environment or attitudes, by qualitatively exploring the processes and experiences of travel behaviour change. These include individuals’ motivations for selecting travel modes, which may be missed in quantitative studies. There is growing recognition that complex public health problems require qualitative as well as quantitative methods, in order to describe and understand communities and learn how to improve and maintain health [25]. Strategies to promote active commuting require depth and breadth of understanding of people’s motivations for beginning and maintaining active commuting, which qualitative methods are suited to exploring; and incorporating qualitative research can enable a better understanding of causal explanations and processes [26].

The effective application of qualitative methods to the study of travel behaviour has been demonstrated by Pooley and Turnbull [27], who included data from 90 semi-structured interviews when examining modes of travel to work in Britain since 1890. They found that reasons for choosing modes of transport were consistent over time, and that once modes of transport were established, people were reluctant to change. Their study highlights the power of qualitative research to offer valuable insights into motivations for travel behaviour.

This paper reports on a qualitative interview study which aimed to explore experiences and processes of selection and change of travel modes for commuting, focusing on the period of home or work relocation; and to consider the theoretical and applied implications for the habit discontinuity and residential self-selection hypotheses.

## Methods

Participants were recruited from the Commuting and Health in Cambridge study cohort. Full details of the recruitment and sampling methods are described in detail elsewhere [28]. In brief, the study examines travel behaviour in adults working in the city of Cambridge, UK, and living within a radius of approximately 30 kilometres. At the same time of year in 2009 (baseline) and 2010 (follow-up) participants completed an extensive questionnaire detailing sociodemographic information regarding themselves and their co-residents; their place of work and residence; their usual mode of travel to work; and other information. Ethical approval was granted by the Hertfordshire Research Ethics Committee.

For this qualitative study, quantitative questionnaire data were used to select a purposive sample. Participants were identified who had relocated home, workplace or both between 2009 and 2010. Those whose relocation was short were assumed to have the least potential for travel behaviour change, and were therefore excluded. A short relocation was defined as one within the same postcode sector, a unit of UK postal geography based on geographic area. While the geographical size of postcode sectors is variable, they comprise about 3,000 addresses on average [29]. Relocation within a postcode sector is therefore likely to involve a relatively short distance, except in very sparsely populated rural areas, and therefore offered a simple and acceptable method of limiting our purposive sample to participants whose relocation was sufficiently long to prompt them to reconsider their commute. From these, a sample reflecting a range of ages, household compositions, work and home locations and travel modes at baseline, and including both men and women, was selected and invited to participate. Participants were invited in rounds, and recruitment continued until saturation of themes was reached. Selected participants were sent an information sheet and those who agreed to participate gave their written informed consent before being interviewed. For each participant at each time point, commute distances along roads between home and work were calculated in an internet mapping browser (Google Maps) using their home and work postcodes as reported in questionnaires, in order to estimate differences in commute distances before and after relocation.

Interviews took place in spring 2011 (between one and two years after relocation), at participants’ homes or workplaces as preferred. Interviews were semi-structured and explored participants’ experiences of and reasons for relocating; their priorities in choosing the new location; how they travelled to and from work before and after relocation and why; and the reasons for any changes (or not) in travel behaviour. Interviews were conducted and analysed by CJ. They were digitally recorded and transcribed verbatim, and the transcripts were checked against the audio recordings. Participants were given pseudonyms.

Qualitative data were analysed and interpreted iteratively in accordance with the grounded theory approach. Grounded theory is a widely used research method in social sciences for the analysis of qualitative data without preconceived theories or hypotheses: analysis is driven by the data so that conclusions and theories emerge from and are grounded in the data [30]. Throughout the conduct of the interviews and upon listening to the audio recordings, codes were developed by CJ according to the topics and responses which arose frequently and were salient to the aims of the study. Segments of the transcripts were assigned to these codes by CJ: codes were not exclusive so that the same segments of transcripts could be assigned to multiple codes. Codes were then grouped into broader themes. DO validated the codes to be used and the assignment of transcript segments to codes. An interim descriptive account was discussed between the authors and with other members of the Commuting and Health in Cambridge study team to validate the emerging findings.

## Results

Questionnaires were completed at both baseline and follow-up by 690 participants. According to home and work postcodes, 126 (18%) of the sample relocated (43 relocated work, 76 relocated home, and 7 relocated both home and work). Twenty-seven relocators had moved within the same postcode sector and were excluded from consideration. Of the remaining 99, all participants whose commuting distance had decreased following relocation were selected because they were few in number (n = 19); and a purposive sample of those whose commute distance had increased was selected according to demographic characteristics, as described above. In total 51 participants were invited to participate and 26 (51%) consented.

Mean commute distance was 7.6 miles at baseline and 11.5 miles at follow-up. One participant commuted the same distance before and after relocating; 14 participants had a longer commute at follow-up and 11 had a shorter commute. Interviews revealed that participants travelled to work using the same mode(s) as they used to travel from work, for the same reasons. When presenting the results, we have therefore not distinguished between journeys to and from work (referring to both as the journey to work).

The participants are profiled in Table 1. Eight participants lived alone, 11 lived with one or more other adults, and seven lived with a partner and children. As can be seen from the participant profiles, many participants had changed their usual mode of commuting following relocation, which provided for rich qualitative data regarding the process of travel behaviour change. Through the conduct of the interviews and qualitative analysis, three main themes were identified which reflected the aim of understanding how travel behaviour changes and how active commuting might be promoted: planning the new journey to work; timing of change in commute mode; and motivation for changing commute mode. These themes are developed below, and are followed by an illustrative case study.

**Table 1:** Profiles of participants (alphabetical order of pseudonyms)

**Pseudonym**	**Age (years)**	**Description of commute before relocation**	**Description of commute after relocation**
Alice	33	Drove to the park-and-ride, then took the bus	Does the same from her new home, which is a similar distance from work
Andy	31	Cycled to the station (15 minutes), took the train to Cambridge and cycled to work (10 minutes). For the first two years in this house he drove to work, before beginning to take the train	Does the same (cycle, train, cycle) from his new house, which is a similar distance from work
Anna	40	Drove most of the way to work, parked in a residential area and then walked the remaining distance to work (around 10 minutes)	Does the same from her new home
Beth	40	Drove to the park-and-ride, and then walked from there to work	Does the same from her new home, which is further from work
Chloe	32	Lived close to work and cycled five minutes to get there	Walks 10 minutes to the bus stop, and takes the bus to work
Chris	31	Cycled seven miles to work on two or three days each week, and drove on the other days	Lives too far from work to cycle and drives every day
Clare	30	Cycled to work	Lives closer to work and uses a scooter
Daniel	45	Drove and occasionally cycled	Still drives to work from his new home, which is closer to work
Dave	25	Walked to work; has also used the bus	Walks to work (45 minutes). He has signed a contract for a new flat, and will soon be moving home much closer to work
Deborah	42	Cycled	Still cycles. The new commute is longer (25 minutes)
Emily	34	Cycled	For her new job she walks to work (20 minutes)
Emma	30	Took the bus, or occasionally drove	Cycles to work (15–20 minutes) from her new home, which is closer to work
Gemma	37	Cycled to work with her children in a trailer	New commute is too far to cycle, especially with the children, so she now drives
George	27	Cycled (15 minutes)	Either walks (12 minutes) or cycles (six minutes); his new home is closer to work
Grace	57	Either cycled or walked (40 minutes)	New home is further from work. She walks to the station (15 minutes), takes the train to Cambridge, then cycles (10 minutes) to work
Jack	49	Cycled	Still cycles; the new journey is slightly longer
Kate	52	Walked	Still walks; the duration of the journey is similar (20 minutes)
Laura	29	Cycled	Her new job is further from home and she needs her car for work, so she now drives to work each day
Liz	46	Drove	She drives to work (lives closer to work)
Lucy	30	Cycled (40 minutes)	Cycles (20 minutes)
Megan	48	Drove to the park-and-ride, and then walked to work	Walks (five minutes)
Mike	55	Motorbike	Walks to the station (15 minutes), takes the train to Cambridge, and then walks to work (15 minutes)
Rebecca	41	Drove	She drives to work (new home is closer to work)
Sophie	28	Cycled four days a week and ran one day a week	She now works in London; she walks to the station (eight minutes), takes the train to London, then walks to work (10 minutes)
Susan	41	Combination of walking and cycling	She still walks (22 minutes) or cycles a similar distance to her new job. She is in the process of buying a new house further from work, and plans to continue cycling or to use public transport
Zoe	37	Used to live in her own home; then she moved in to her mother’s house (further from work) to care for her when she became ill, and drove to work	She is now living back at her own home again and either drives or cycles to work

### Planning the new journey to work

When making decisions regarding relocation, there was a complex interplay of factors to be considered, including distance to family and friends, distance to amenities (shops, leisure), desirability of the area, needs of family members (including distance to local schools), and the availability and cost of housing, as well as commute distance (sometimes for multiple members of the household). Changing the commute to work was not a primary motivation for relocating for any participant; but once the decision to relocate had been made and possible new locations were being considered, then participants described varying amounts of consideration or importance attributed to the commute to work.

At one extreme, some participants did not consider the commute to be a salient priority at all when relocating:

*“The place of work, I really haven’t used location as a criteria for taking or applying for a job, so that’s something that I tend to just deal with as it needs dealing with, rather than using that as a factor to influence where I apply to.” (Emily, moved workplace)*.

Generally, however, the commute was considered to be an influential factor to some extent. Different aspects of the commute were thought to be important. Some participants prioritised the convenience and speed with which they could get to work, regardless of mode:

*“As long as I found a way that was relatively cheap and easy to get to work, I wouldn’t mind too much what it was.” (Anna, moved home)*.

Fewer prioritised the particular mode of travel by which they would commute from their new location:

*“It was one of the things that attracted me to applying for the job here was that I could still cycle it.” (Jack, cycled to work both before and after moving workplace)*.

*“One of the attractions of the job, you know, was, included that it’s on a bus route and actually it’s easy cycling.” (Susan, cycled or walked to work both before and after moving workplace)*.

It was relatively easy for participants to find a location to suit their preference for commuting by car or public transport; in contrast, participants who preferred to commute by bike or on foot were not always able to ensure this. The main reasons for moving home were to buy a first house or a larger house, and decreasing house prices further from Cambridge city centre (where all of the participants worked) meant that this frequently necessitated moving further from work. Indeed, more participants had a longer commute following relocation than a shorter commute. Consequently, it was often not possible to walk or cycle for the entire journey. Hence whilst some participants would have preferred to relocate to an area from where they could walk or cycle to work, this preference often lost out to competing priorities. Participants who were not able to maintain their preference for active commuting felt they experienced a “loss” or a “sacrifice”:

*“I think with every move there’s a sacrifice along the way and it’s the how I get to work that’s sacrificed really.” (Gemma, cycled before moving and drove after moving home further from work)*.

*“I’ve lost the cycling… I think it was the right thing for me to do in terms of my career, that side of it, and I think that’s slightly more important than the cycling, the commute.” (Laura, cycled before moving and drove after moving workplace further from home)*.

Ensuring that walking or cycling to work was possible from their new location was described as preferable or important only by participants who had commuted actively from their previous location; those who had commuted by non-active modes before relocation did not prioritise walking or cycling for their new locations.

A factor related to the commute which was more commonly adhered to when selecting a new location was the distance between home and work. Some participants described searching within a radius. Maximum tolerable commute distances varied, and were described in terms of distance or time:

“I did sort of do a kind of within a fifteen to twenty miles radius around [work]” (Anna, drove to work before and after moving home).

*“I think I just had an idea of how long I was willing to travel. I think half an hour is a good length of time” (Jack, cycled to work before and after moving workplace)*.

Related to the importance attributed to the commute when making decisions regarding relocation was the knowledge which people had about their new commute before they relocated. Participants who considered the commute in detail, and prioritised aspects of the commute (such as distance, convenience or mode) necessarily had knowledge of their new commute before relocating. Knowledge of how they would commute from their new location was gained before they moved by talking to other people, rehearsing the new journey, and building upon previous experience of the local area:

“I did the, a few trial runs and the main consideration was time and it didn’t seem to take too long… we did it first and we’ve got friends who live there, so got their experience as well on that.” (Chris, moved home).

“I had cycled there before just sort of exploring the area but I think I did try the route out explicitly when we were looking at thinking about buying it.” (Deborah, cycled before and after moving home).

In contrast, other participants described having only vague plans about how they would commute from their new location before the move took place:

“I didn’t look at how far I would be, I didn’t know.” (Clare, moved home).

“I didn’t know the area at all, I knew there must be a bus and I suppose I knew there would be a way I could get to work but I didn’t really consider it too much when we were moving, I knew I’d be able to sort it out.” (Chloe, moved home).

### Timing of change in commute mode

Participants changed or adjusted their mode of travel to work at different stages throughout relocation. Some initiated a change in commute mode immediately; others trialled different modes before deciding which was preferable, or adjusted their travel behaviour subsequently following relocation.

Participants with only vague plans for their new commute before they actually relocated (such as Clare and Chloe, see above) necessarily had to formulate ideas and plans for the commute after moving. In addition, participants who had detailed knowledge of, and had rehearsed, the new commute before relocating also found themselves adjusting their new commute again after the move took place. Further adjustments included taking different routes by bike, departing from home in the car at different times in the morning to avoid traffic, or taking trains at different times:

*“I’ve changed the way I come to work because my original journey used to take a long time, stuck in traffic jams, so I’ve tried it a few times in different directions” (Gemma, began driving after moving home)*.

Furthermore, some participants used a certain mode of travel immediately after relocation and later completely altered their initial decision. Two participants initially commuted solely by motor vehicle following relocation (inactive commuting), then after experiencing the new commute switched to active commuting:

*“I still like for the first few months just got the bus. I mean I kind of knew the bus route, I knew that there were bus routes and things, so no I didn’t think about cycle routes or anything like that. I just, I assumed that initially I would just get the bus, but probably like after starting to do that for a while and then realising it’s not that far away… that probably influenced me, yeah, to start thinking a bit more about getting a bike.” (Emma, moved home. Her initial commute by bus did not include any active travel because the bus stops were close to her home and workplace)*.

“I used to drive, um, first two years that I worked here and I hated it, it’s just horrible, it’s just constant traffic jams… I wasn’t really sure what to do about it but, like I say, I spent two years doing that. [Then] I just thought I’d just had enough of taking the car so I’d give it a go on the train.” (Andy. His train journey was defined as active commuting because it was combined with cycling. Note that this participant was describing a home relocation and travel behaviour change prior to baseline; he also moved home between baseline and follow-up and continued to commute by a combination of public transport and cycling).

The time elapsed between relocating and changing commute modes is striking here. The changes in modes experienced by these participants occurred two months and almost two years after relocating, respectively.

### Motivation for changing commute mode

Regarding their commute since relocation, participants described the travel modes they had chosen as the most convenient, cheapest, quickest, and most reliable. These were the primary motivating factors for choosing travel modes, whether active or motorised modes, and were the factors most commonly cited by participants; they were discussed first, with consideration to physical activity only being given afterwards. The following quotes illustrate participants being motivated by these factors to commute actively (whether initiating or maintaining active commuting following relocation):

“*I don’t actually enjoy cycling really, I do it purely because it’s convenient, like that really is the only motivation I think”. (Emma, took the bus before moving home, and after moving began cycling to work – after initially taking the bus, as described above).*

“I could drive here but then I have to park somewhere… I suppose I could get the bus but it would probably take as long to come by bus as it would for me to walk anyway… yes, so walking is the most practical way.” (Kate, walked both before and after moving home).

“I don’t really want to drive to work, the parking is a pain and, you know, it would probably be slower than the bike most days.” (Deborah, cycled both before and after moving home).

“It’s just, it is the reliability of it, I know that it will take me 20 minutes whereas other ways it’s a bit not sure, also my work doesn’t really have any parking so it’s just more convenient.” (Lucy, cycled to work before and after moving home).

“The nice thing about walking and cycling is that you set your own timetable…” (Susan, used a mixture of walking or cycling both before and after moving workplace).

“I think the main reason would be that it’s cheap, that it’s reliable, you can, that it’s in town as fast as other ways of transport, well faster. And you’re flexible as well and you don’t need a parking space which is a Cambridge problem I guess.” (George, cycled to work before moving home, and after moving used a mixture of walking and cycling).

The extra physical activity obtained by commuting actively was not described by any participants as a primary motivating factor to begin active commuting. Physical activity during active commuting was incidental, and usually unanticipated:

“I used to cycle every day and it does, it gives you higher fitness levels without, you know, you really thinking too much about it. [Interviewer: Was that the main reason for bicycling then, the fitness?] No, it was free!… cost I suppose, and traffic, you know, you don’t want to sit in traffic when you can just cycle to work, it’s easier.” (Chris, cycled before moving home, and after moving began to drive).

Although active commuting was not *initiated* primarily for health reasons, once participants had begun commuting actively for other reasons then the health benefits were sometimes recognised:

“I think I’ve got fitter and so I cycle quicker now.” (Emma, took the bus before moving home, and after moving began to cycle).

This motivated some participants to *maintain* active commuting if possible when relocating, as described above.

It emerged from the interviews that using public transport was an important way of incorporating walking or cycling in the journey to work. Participants were motivated to commute by public transport by similar considerations of convenience, reliability, speed and cost:

“Well I won’t get parking, it’s busy, I don’t see the point in driving that distance unnecessarily really and it costs money.” (Chloe, used to cycle to work, and after moving home began taking the bus).

Public transport commuting usually entailed some active travel (walking or cycling to and from bus stops or railway stations). This gain in daily physical activity was also incidental rather than intended — as illustrated by the case of Andy, described above, who changed from car commuting to train and bike commuting almost two years after relocating.

Another way in which participants incidentally incorporated walking or cycling in their journey was by using park-and-ride sites – facilities whereby drivers leave their cars in a free car park on the outskirts of a city and transfer to public transport (usually bus) to complete their journey into the city. Although park-and-ride users are generally expected to transfer to public transport, some participants described parking at a park-and-ride site and then walking or cycling for the last section of the journey rather than taking the bus. Again, the motivations for walking or cycling for this section of the journey remained the same (speed, cost, convenience and reliability), and the physical activity involved was not a motivating factor:

“I tend to walk in then because I hate this last final queue of traffic, you know, just from [the park-and-ride] into here because it’s just a standstill all the way in, so I always walk it instead… if I was to take the bus it’s almost the same price as parking [at work] so it defeats the purpose then you know.” (Beth, moved home).

“The buses were really convenient, they would go maybe every five minutes, but then there would be a big queue of traffic probably, you know, and I’d be quicker walking past the queue.” (Megan, used to drive to the park-and-ride and then walk to work before moving home, and after moving closer to work began walking the entire journey).

Four participants described parking at a park-and-ride site and then walking the remaining distance (either at baseline, follow-up or both). Another participant parked in a residential area almost a mile from her workplace and then walked to work:

“Because I want to get parked without any cost because the car parking charges on-site are very high over the period of a year” (Anna, both before and after moving home she drove to the same residential area to park and then walk to work).

This again demonstrates the influence of cost in motivating people to include walking as an incidental component of their daily commute.

### Case study

A case study illustrates how these themes apply to a single participant. Mike, a man in his fifties, moved home from a town eight miles from Cambridge city centre to a town 16 miles from Cambridge city centre. From his first location he travelled to work using a motorbike; upon relocating, he started using the train to commute to work:

“So it’s about a fifteen minute walk to the railway station and then catch the train into Cambridge, then it’s about another fifteen minutes from the railway station to here. So it’s walk, train, walk… The train works out cheaper and it’s also quicker. Because parking is difficult in the centre of Cambridge, on this site in particular, it would be difficult to bring the car in and it’s slower and more expensive so yeah, that’s the main reason… Before I lived there I would come in on the motorbike… That was quite good but really it’s just more convenient on the train… It’s a major consideration, where I live to how easy it is to commute in… [When I moved home] I was intending to use the train… But I was surprised how good it was. It’s excellent… Because I think it’s sometimes difficult to fully work out how convenient places are going to be to live until you’re actually there, that’s when you find the problems. You know, it could be just a very unreliable line, some of the train lines are more unreliable than others and you don’t really know that until you start using them regularly… It’s nice if you can get the seat on the train. I have actually changed, I used to catch a slightly later train which you’re guaranteed a seat on but that’s the school train so… that’s quite noisy and full by the time it arrives… But it gets in, yeah, about twenty minutes later… I think my health has improved since I’ve been doing the regular walking, yes. Yeah. Yes. Certainly weight-wise. (Laughs) Yeah, I think I have become a bit fitter, certainly since I’ve been doing all the regular walking, yes.”

This man prioritised his commute to work when selecting his new location; similarly to other participants, he considered the “ease” of getting to work, rather than preferring a particular mode. He made plans to commute by train before he relocated, but made some subsequent adjustment by changing to a train at a different time. He explained the reason for this adjustment – that “it’s difficult to fully work out how convenient places are going to be to live until you’re actually there” – which may also explain why other participants adjusted their commute slightly, or totally, despite having had knowledge and plans for their new commute before relocating. This man’s reasons for changing to the train were that “it’s cheaper and it’s also quicker”, in keeping with the overall motivating factors when selecting travel modes being cost, convenience, speed and reliability, described above. He experienced an incidental increase in physical activity associated with the commute, from zero to 60 minutes per day. This increase in physical activity was unanticipated, but he recognised increased fitness and weight loss after commencing his new commute. His commute distance increased following relocation, but public transport enabled him to incorporate physical activity into his commute.

## Discussion

### Motivations for active commuting, and incidental physical activity

These accounts of working adults in Cambridge, UK, who had relocated home or workplace in the previous one to two years, demonstrate that commuters are motivated by convenience, cost, speed and reliability when selecting commute modes around the period of relocation. Health consequences were not motivating factors; participants who began commuting actively experienced an *incidental* increase in physical activity in the commute. Impacts on physical activity levels and fitness were neither intended nor anticipated, although they were often recognised and welcomed once active commuting commenced. Pooley and Turnbull [27] interviewed Britons about their travel to work since 1890, and uncovered similar motivations of low cost, speed and convenience; and in a survey study by Kingham et al. [2], employees reported willingness to consider cycling and public transport rather than the car if frequency, reliability and convenience were improved and discounted travel were offered (amongst other factors). Emphasising the relative convenience, cost, speed and reliability of active commuting, rather than health benefits, may be most effective in promoting walking and cycling to work.

In addition to emphasising these relative benefits of active commuting (thereby influencing public perceptions), direct alterations to provision should be considered. Previous studies have demonstrated that modifying the cost of travel options, for example by providing temporarily subsidised or free public transport [31], influences travel mode choice. This study suggests that as well as cost, direct alterations to the relative speed, convenience and reliability of active commuting and public transport with respect to car travel should be considered in an effort to increase rates of active commuting.

Although health benefits did not motivate people to *initiate* active commuting, participants who experienced and recognised the health benefits of doing so were sometimes subsequently motivated to *maintain* active commuting. It was only participants who commuted actively from their previous location who described a preference for active commuting at their new location. This preference was not upheld when competing priorities prevented people from being able to select a location from where they could commute by bike or on foot, mainly due to distance. Public transport or park-and-ride sites could enable active commuting for people in this situation (see below).

### Public transport and park-and-ride

Travelling by public transport led to incidental increases in physical activity in the daily commute. Previous research has found that public transport users can achieve recommended daily levels of physical activity through active travel alone, and that public transport commuters are more physically active overall and have lower levels of obesity than car commuters [32-35]. Hence providing convenient, reliable and affordable public transport shows promise as a strategy for (incidentally) increasing daily physical activity levels. The present study adds to existing literature by describing the factors which motivate people to begin commuting by public transport.

The cost of parking at work, and the relative speed of walking rather than taking the bus from park-and-ride sites, motivated people to park for free at park-and-ride sites and then walk to work. This again demonstrates incidental physical activity associated with commute choices driven by speed, convenience and cost. Previous research has found that free parking at work is a predictor of car commuting, and that fewer people drive to work when there are parking charges [36-38]. This study suggests that if people are able to park for free within walking or cycling distance of work then they will be motivated to do so if it is the quickest, cheapest, and most convenient option.

These strategies of incorporating walking or cycling into a longer journey to work highlight a dichotomy regarding the influence of public transport on active commuting and physical activity. Good public transport services can either increase physical activity (by competing with car commuting) or decrease physical activity (by competing with walking or cycling) [22]. Some relocators in this sample — such as those who replaced car commuting with train commuting combined with walking or cycling — incidentally increased their physical activity as a result of using public transport; others experienced greater physical activity due to the lack of superior public transport options, for example when choosing to walk from park-and-ride sites to work rather than taking the bus.

The use of public transport and park-and-ride facilities enabled participants to incorporate incidental walking or cycling into their commute when it was too far to walk or cycle for the entire journey. This is especially important since more participants had a longer than a shorter commute following relocation. Indeed, previous studies have reported that many people live too far from work to be able to walk or cycle there [2]: according to the 2009 National Travel Survey, the average length of a commuting trip in the UK was 8.6 miles, with 80% of respondents commuting two miles or more per trip [39]. Accordingly, distance is commonly cited as a barrier to active travel [10,16]. This study demonstrates that public transport and park-and-ride sites enable some commuters to initiate or maintain active travel despite an increase in overall commute distance. Although facilitating active travel is not always an aim of park-and-ride provision [40], allowing or even promoting walking or cycling from park-and-ride sites, or providing other free parking within walking or cycling distance of employment sites, should be considered as a strategy to promote everyday physical activity.

These accounts highlight that it is not straightforward to define commuting journeys as being ‘walkable’ or ‘bikeable’, because journeys can be made in multiple stages of which only part need be of a ‘walkable’ or ‘bikeable’ distance. Distance need not be an obstacle to active commuting, and people living in rural locations can include walking or cycling in their journey to work in combination with using public transport or park-and-ride sites.

### Residential self-selection and habit discontinuity hypotheses

Some relocators had only vague plans about how they would commute before they relocated, and were susceptible to changes in commute mode following relocation. Others had detailed knowledge and had planned their new commute in detail before they relocated, yet still adjusted their intended commute behaviour slightly or totally once in their new location. This illustrates that participants even with strong plans may be open to changing their commuting behaviour following relocation.

These accounts challenge the straightforward self-selection hypothesis, which suggests that people relocate to areas from where they will be able to commute in their preferred or expected way, and then do so. First, some participants described a preference for active commuting, but were unable to realise this because of competing priorities, specifically the wish to relocate further from the city centre to buy a first or a larger house. Second, in this sample, even participants who had expectations and plans for their new commute were amenable to changes in travel behaviour following relocation.

Change in commute mode occurred for some participants months after relocation, rather than immediately. This suggests that there may be an extended ‘window of opportunity for behaviour change’ (described by the habit discontinuity hypothesis) associated with context change, and that strong, irreversible habits are not necessarily formed immediately following relocation, even in participants who have strong intention regarding how they will commute. These non-immediate changes in travel behaviour may be missed in quantitative surveys assessing travel behaviour at single points before and after relocation. Furthermore, some participants considered their travel options when relocating, and selected the same mode of commuting at their new location. Thus their travel behaviour before and after relocation remained the same, not because they had simply continued their habitual behaviours and failed to reconsider the alternatives, but because they had re-selected the same mode for similar reasons as pertained to their previous commuting journey. Again, this reassessment would be missed in simple comparisons of travel behaviour before and after relocation.

These results suggest that a straightforward interpretation of the self-selection hypothesis is too deterministic, and does not reflect the constant negotiation, reassessment and trialling experienced by our participants upon relocating. This reminds us that commuters do not necessarily simply decide how they prefer to travel, move to an area to facilitate this, and then travel in their expected way.

The context change of relocation appears to provide an extended window of opportunity for behaviour change since some participants changed their travel behaviour weeks or months after relocation. For some, therefore, commuting *habits* (which are resistant to change) are not formed during this period. For these commuters — even those who had strong intentions and plans for how they would commute before they relocated — interventions to promote active commuting may therefore be effective for a considerable period after relocation.

### Limitations and strengths

The limitations of this study are inherent in its qualitative design. Participants were purposively sampled, and the sample is not representative of relocators or commuters across the UK. For example, the prevalence of active commuting and the average socioeconomic status of participants in the Commuting and Health in Cambridge study cohort are above average. Furthermore, analysis was guided by the data and by the lead researcher. However, qualitative research does not aim to be generalisable or representative; it focuses on in-depth, exploratory accounts in a particular setting in order to gain deeper and broader understanding of an issue, which we consider this study to have been successful in achieving.

Participants were excluded from the study if they had relocated within the same postcode sector, on the assumption that their distance of relocation was short. Since relocation distance was not calculated for all relocators before applying this exclusion criterion, it is possible that some participants who had relocated a short distance were eligible for inclusion, while others who had relocated a greater distance were excluded. However, we regarded this as an acceptable method for purposively sampling participants whose relocation was likely to have been sufficient to prompt (re)consideration of their commuting behaviour.

Commute distances before and after relocation were estimated using road networks. Such estimates, which assume that all participants travelled along roads and along the most direct route, are not necessarily precise estimates of the actual distances followed by individuals in practice. However, precisely estimating these distances was not an aim of the study.

The strengths of this study again lie in its qualitative design, which enabled in-depth insight into how and why people begin and maintain active commuting, and has implications for policy and practice. This study has added a layer of context and understanding to the field of active travel research currently dominated by quantitative methods, and has highlighted the power of qualitative data to expand upon and challenge our knowledge of travel behaviour. It has uncovered commuters’ attitudes and motivations in a way which is not possible using quantitative techniques, clearly elucidating areas for further research or intervention which have the potential to improve levels of active commuting and physical activity.

Using the period of relocation to explore travel behaviour was a further strength of this study, because it enabled insight into the processes of behaviour change. Commuting is habitual and relatively resistant to spontaneous change: in the absence of experimental interventions to induce behaviour change, relocation is a type of ‘natural’ or proxy intervention which breaks the commuting habit and makes behaviour change more likely. Identifying relocators enabled an examination of travel motivations at the time of relocation, when travel behaviour is more likely to be considered, and therefore strengthened this investigation into what motivates commute choices.

## Conclusion

Efforts to promote active commuting may be most effective not when emphasising the potential health benefits, but by improving and emphasising the relative convenience, cost, speed and reliability – at least around the period of relocation. Improving and promoting these factors for public transport and providing cost-free parking at a walkable or bikeable distance from major sites of employment show promise for increasing active commuting for the large number of people living too far from work to be able to walk or cycle for the entire journey, and should be considered as strategies to (incidentally) improve physical activity levels. Accordingly, overall distance from home to work should not be regarded as a barrier to active commuting. There may be an extended window of opportunity for travel behaviour change following context change, and initial plans or intentions regarding travel behaviour are malleable and susceptible to adjustment or total change during this period. Efforts to promote active travel may therefore be effective if applied following context changes such as relocation.

## Competing interests

There are no competing interests to declare.

## Authors’ contributions

CJ conceptualised and designed the study; collected, analysed and interpreted the data; and drafted the paper. DO also conceptualised the study, assisted in data interpretation, and critically revised the paper. Both authors read and approved the final manuscript.
